# 5-(4-Phenoxy­phen­yl)-1,3,4-thia­diazol-2-amine

**DOI:** 10.1107/S1600536809013257

**Published:** 2009-04-18

**Authors:** Rong Wan, Yao Wang, Feng Han, Peng Wang

**Affiliations:** a Department of Applied Chemistry, College of Science, Nanjing University of Technology, No. 5 Xinmofan Road, Nanjing, Nanjing 210009, People’s Republic of China

## Abstract

The title compound, C_14_H_11_N_3_OS, was synthesized by the reaction of phenoxy­benzoic acid and thio­semicarbazide. The thia­diazole ring makes dihedral angles of 0.99 (16) and 86.53 (18)°, respectively, with the benzene and phenyl rings. The dihedral angle between the benzene and phenyl rings is 87.17 (19)°. Intra­molecular C—H⋯S contacts are present. In the crystal, inter­molecular N—H⋯N hydrogen bonds link the mol­ecules.

## Related literature

For the fungicidal and herbicidal activities of thia­diazole derivatives, see: Chen *et al.* (2000 ▶); Kidwai *et al.* (2000 ▶); Vicentini *et al.* (1998 ▶). For their insecticidal activities, see: Arun *et al.* (1999 ▶); Wasfy *et al.* (1996 ▶). For bond-length data, see: Allen *et al.* (1987 ▶).
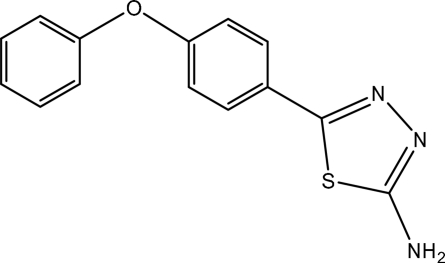


## Experimental

### 

#### Crystal data

C_14_H_11_N_3_OS
*M* *_r_* = 269.32
Monoclinic, 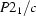

*a* = 13.409 (3) Å
*b* = 10.582 (2) Å
*c* = 9.5710 (19) Å
β = 108.58 (3)°
*V* = 1287.3 (4) Å^3^
*Z* = 4
Mo *K*α radiation
μ = 0.25 mm^−1^
*T* = 293 K
0.30 × 0.20 × 0.20 mm

#### Data collection

Enraf–Nonius CAD-4 diffractometer
Absorption correction: ψ scan (North *et al.*, 1968 ▶) *T*_min_ = 0.930, *T*_max_ = 0.953
2438 measured reflections
2336 independent reflections
1596 reflections with *I* > 2σ(*I*)
*R*_int_ = 0.032
3 standard reflections every 200 reflections intensity decay: 1%

#### Refinement

*R*[*F* ^2^ > 2σ(*F* ^2^)] = 0.051
*wR*(*F* ^2^) = 0.141
*S* = 1.00
2336 reflections
172 parameters
H-atom parameters constrained
Δρ_max_ = 0.22 e Å^−3^
Δρ_min_ = −0.22 e Å^−3^

### 

Data collection: *CAD-4 EXPRESS* (Enraf–Nonius, 1989 ▶); cell refinement: *CAD-4 EXPRESS*; data reduction: *XCAD4* (Harms & Wocadlo,1995 ▶); program(s) used to solve structure: *SHELXS97* (Sheldrick, 2008 ▶); program(s) used to refine structure: *SHELXL97* (Sheldrick, 2008 ▶); molecular graphics: *SHELXTL* (Sheldrick, 2008 ▶); software used to prepare material for publication: *SHELXL97*.

## Supplementary Material

Crystal structure: contains datablocks global, I. DOI: 10.1107/S1600536809013257/at2760sup1.cif

Structure factors: contains datablocks I. DOI: 10.1107/S1600536809013257/at2760Isup2.hkl

Additional supplementary materials:  crystallographic information; 3D view; checkCIF report

## Figures and Tables

**Table 1:** Hydrogen-bond geometry (Å, °)

*D*—H⋯*A*	*D*—H	H⋯*A*	*D*⋯*A*	*D*—H⋯*A*
N3—H3*A*⋯N2^i^	0.86	2.21	3.042 (4)	162
N3—H3*B*⋯N1^ii^	0.86	2.26	3.094 (3)	163
C9—H9*A*⋯S	0.93	2.72	3.133 (4)	108

Symmetry codes: (i) 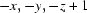; (ii) 
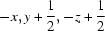
.

**Fig. 1.**
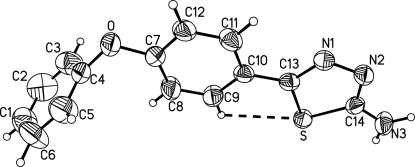
A view of the molecular structure of (I). Displacement ellipsoids are drawn at the 50% probability level. Dashed lines indicate the intramolecular C—H···S contact.

**Fig. 2.**
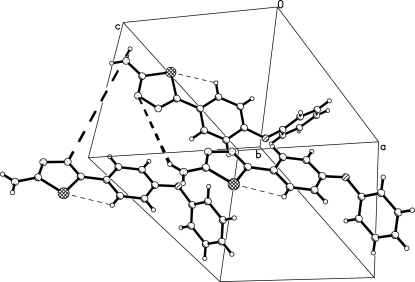
Partial packing view showing the hydrogen-bonded network. Dashed lines indicate intermolecular N—H···N hydrogen bonds.

## supplementary crystallographic information

### Comment

Thiadiazole derivatives containing the thiazolidinone unit are of great interest because of their chemical and pharmaceutical properties. Some derivatives have fungicidal and herbicidal activities (Chen *et al.*, 2000; Kidwai *et al.*, 2000; Vicentini *et al.*, 1998); some show insecticidal activities (Arun *et al.*, 1999; Wasfy *et al.*, 1996). We report here the crystal structure of the title compound, (I).

The molecular structure of (I) is shown in Fig.1, in which the bond lengths (Allen *et al.*, 1987) and angles are generally within normal ranges. The thiadiazole ring makes dihedral angles of 0.99 (16)° and 86.53 (18) ° with the benzene and phenyl rings, respectively. The dihedral angle between the benzene and phenyl rings is 87.17 (19)°. There are intramolecular C—H···S contacts (Fig. 1), and intermolecular N—H···N hydrogen bonds, linking the molecules into chains along the *b* axis (Fig. 2).

### Experimental

Phenoxybenzoic acid (5 mmol) and thiosemicarbazide (5 mmol) were added in toluene (50 ml), which is heated under reflux for 4 h. The reaction mixture was left to cool to room temperature, poured into ice water, filtered, and the filter cake was crystallized from acetone to give pure compound (I) [m.p. 513–514 K]. Crystals of (I) suitable for X-ray diffraction were obtained by slow evaporation of an acetone solution.

### Refinement

All H atoms were placed geometrically at the distances of C—H = 0.93 Å and N—H = 0.86 Å, and included in the refinement in riding motion approximation with *U*_iso_(H) = 1.2*U*_eq_ of the carrier atom.

### Crystal data

**Table:**

C_14_H_11_N_3_OS	*F*(000) = 560
*M**_r_* = 269.32	*D*_x_ = 1.390 Mg m^−^^3^
Monoclinic, *P*2_1_/*c*	Melting point: 542 K
Hall symbol: -P 2ybc	Mo *K*α radiation, λ = 0.71073 Å
*a* = 13.409 (3) Å	Cell parameters from 25 reflections
*b* = 10.582 (2) Å	θ = 10–13°
*c* = 9.5710 (19) Å	µ = 0.25 mm^−^^1^
β = 108.58 (3)°	*T* = 293 K
*V* = 1287.3 (4) Å^3^	Block, colourless
*Z* = 4	0.30 × 0.20 × 0.20 mm

### Data collection

**Table:**

Enraf–Nonius CAD-4 diffractometer	1596 reflections with *I* > 2σ(*I*)
Radiation source: fine-focus sealed tube	*R*_int_ = 0.032
graphite	θ_max_ = 25.3°, θ_min_ = 2.5°
ω/2θ scans	*h* = 0→16
Absorption correction: ψ scan (North *et al.*, 1968)	*k* = 0→12
*T*_min_ = 0.930, *T*_max_ = 0.953	*l* = −11→10
2438 measured reflections	3 standard reflections every 200 reflections
2336 independent reflections	intensity decay: 1%

### Refinement

**Table:**

Refinement on *F*^2^	Primary atom site location: structure-invariant direct methods
Least-squares matrix: full	Secondary atom site location: difference Fourier map
*R*[*F*^2^ > 2σ(*F*^2^)] = 0.051	Hydrogen site location: inferred from neighbouring sites
*wR*(*F*^2^) = 0.141	H-atom parameters constrained
*S* = 1.00	*w* = 1/[σ^2^(*F*_o_^2^) + (0.074*P*)^2^ + 0.12*P*] where *P* = (*F*_o_^2^ + 2*F*_c_^2^)/3
2336 reflections	(Δ/σ)_max_ < 0.001
172 parameters	Δρ_max_ = 0.22 e Å^−^^3^
0 restraints	Δρ_min_ = −0.22 e Å^−^^3^

### Special details

**Table:**

Geometry. All e.s.d.'s (except the e.s.d. in the dihedral angle between two l.s. planes) are estimated using the full covariance matrix. The cell e.s.d.'s are taken into account individually in the estimation of e.s.d.'s in distances, angles and torsion angles; correlations between e.s.d.'s in cell parameters are only used when they are defined by crystal symmetry. An approximate (isotropic) treatment of cell e.s.d.'s is used for estimating e.s.d.'s involving l.s. planes.
Refinement. Refinement of *F*^2^ against ALL reflections. The weighted *R*-factor *wR* and goodness of fit *S* are based on *F*^2^, conventional *R*-factors *R* are based on *F*, with *F* set to zero for negative *F*^2^. The threshold expression of *F*^2^ > σ(*F*^2^) is used only for calculating *R*-factors(gt) *etc*. and is not relevant to the choice of reflections for refinement. *R*-factors based on *F*^2^ are statistically about twice as large as those based on *F*, and *R*- factors based on ALL data will be even larger.

### Fractional atomic coordinates and isotropic or equivalent isotropic displacement parameters (Å^2^)

**Table:**

	*x*	*y*	*z*	*U*_iso_*/*U*_eq_	
S	0.07308 (7)	0.15388 (7)	0.18224 (8)	0.0444 (3)	
O	0.3343 (2)	−0.0997 (2)	−0.2453 (3)	0.0751 (8)	
N1	0.0991 (2)	−0.0816 (2)	0.2420 (3)	0.0438 (6)	
N2	0.0458 (2)	−0.0359 (2)	0.3336 (3)	0.0450 (6)	
N3	−0.0237 (2)	0.1545 (2)	0.3882 (3)	0.0564 (7)	
H3A	−0.0449	0.1197	0.4550	0.068*	
H3B	−0.0344	0.2337	0.3693	0.068*	
C1	0.4549 (4)	0.1716 (4)	−0.4561 (5)	0.0764 (12)	
H1B	0.4826	0.2314	−0.5046	0.092*	
C2	0.3629 (4)	0.1137 (4)	−0.5265 (5)	0.0870 (13)	
H2B	0.3273	0.1343	−0.6242	0.104*	
C3	0.3208 (3)	0.0249 (4)	−0.4566 (5)	0.0729 (11)	
H3C	0.2572	−0.0142	−0.5061	0.087*	
C4	0.3732 (3)	−0.0048 (3)	−0.3145 (4)	0.0526 (8)	
C5	0.4662 (3)	0.0517 (4)	−0.2422 (4)	0.0721 (11)	
H5A	0.5027	0.0301	−0.1451	0.086*	
C6	0.5062 (3)	0.1415 (4)	−0.3144 (5)	0.0834 (13)	
H6A	0.5691	0.1818	−0.2648	0.100*	
C7	0.2835 (3)	−0.0671 (3)	−0.1459 (4)	0.0501 (8)	
C8	0.2482 (3)	0.0534 (3)	−0.1339 (4)	0.0586 (9)	
H8A	0.2603	0.1185	−0.1918	0.070*	
C9	0.1947 (3)	0.0763 (3)	−0.0349 (4)	0.0537 (9)	
H9A	0.1701	0.1573	−0.0272	0.064*	
C10	0.1769 (2)	−0.0194 (3)	0.0532 (3)	0.0383 (7)	
C11	0.2148 (2)	−0.1399 (3)	0.0400 (4)	0.0481 (8)	
H11A	0.2044	−0.2051	0.0989	0.058*	
C12	0.2673 (2)	−0.1634 (3)	−0.0592 (4)	0.0516 (8)	
H12A	0.2919	−0.2443	−0.0677	0.062*	
C13	0.1199 (2)	0.0043 (3)	0.1577 (3)	0.0375 (7)	
C14	0.0255 (2)	0.0854 (3)	0.3138 (3)	0.0406 (7)	

### Atomic displacement parameters (Å^2^)

**Table:**

	*U*^11^	*U*^22^	*U*^33^	*U*^12^	*U*^13^	*U*^23^
S	0.0627 (5)	0.0291 (4)	0.0463 (4)	0.0030 (4)	0.0244 (4)	0.0026 (3)
O	0.101 (2)	0.0436 (14)	0.115 (2)	−0.0098 (13)	0.0814 (18)	−0.0134 (14)
N1	0.0596 (16)	0.0287 (13)	0.0480 (14)	0.0027 (11)	0.0241 (12)	0.0010 (11)
N2	0.0619 (17)	0.0319 (13)	0.0483 (15)	0.0004 (12)	0.0274 (13)	0.0014 (11)
N3	0.091 (2)	0.0384 (14)	0.0525 (15)	0.0099 (14)	0.0404 (15)	0.0044 (13)
C1	0.085 (3)	0.072 (3)	0.082 (3)	−0.004 (2)	0.040 (2)	0.017 (2)
C2	0.097 (3)	0.081 (3)	0.070 (3)	0.009 (3)	0.008 (2)	0.018 (2)
C3	0.052 (2)	0.066 (3)	0.088 (3)	−0.0005 (19)	0.005 (2)	−0.004 (2)
C4	0.057 (2)	0.0439 (18)	0.069 (2)	−0.0033 (16)	0.0369 (18)	−0.0102 (17)
C5	0.081 (3)	0.084 (3)	0.049 (2)	−0.020 (2)	0.0162 (19)	0.0017 (19)
C6	0.068 (3)	0.095 (3)	0.086 (3)	−0.035 (2)	0.021 (2)	0.006 (3)
C7	0.055 (2)	0.0393 (18)	0.067 (2)	−0.0050 (15)	0.0341 (17)	−0.0090 (16)
C8	0.079 (2)	0.0385 (18)	0.075 (2)	0.0031 (16)	0.049 (2)	0.0069 (16)
C9	0.074 (2)	0.0316 (16)	0.069 (2)	0.0056 (16)	0.0412 (19)	0.0000 (15)
C10	0.0392 (16)	0.0307 (15)	0.0458 (16)	−0.0012 (12)	0.0147 (13)	−0.0015 (12)
C11	0.0474 (18)	0.0367 (17)	0.065 (2)	0.0023 (14)	0.0253 (16)	0.0064 (15)
C12	0.0516 (19)	0.0341 (16)	0.077 (2)	−0.0005 (14)	0.0322 (17)	−0.0031 (16)
C13	0.0410 (16)	0.0299 (14)	0.0392 (15)	−0.0004 (12)	0.0093 (13)	0.0004 (12)
C14	0.0527 (19)	0.0329 (16)	0.0358 (15)	−0.0009 (13)	0.0137 (14)	−0.0029 (12)

### Geometric parameters (Å, °)

**Table:**

S—C14	1.740 (3)	C3—H3C	0.9300
S—C13	1.746 (3)	C4—C5	1.357 (5)
O—C7	1.378 (4)	C5—C6	1.380 (5)
O—C4	1.393 (4)	C5—H5A	0.9300
N1—C13	1.303 (4)	C6—H6A	0.9300
N1—N2	1.383 (3)	C7—C12	1.374 (4)
N2—C14	1.314 (4)	C7—C8	1.378 (4)
N3—C14	1.333 (4)	C8—C9	1.381 (4)
N3—H3A	0.8600	C8—H8A	0.9300
N3—H3B	0.8600	C9—C10	1.386 (4)
C1—C6	1.349 (5)	C9—H9A	0.9300
C1—C2	1.349 (6)	C10—C11	1.393 (4)
C1—H1B	0.9300	C10—C13	1.462 (4)
C2—C3	1.375 (6)	C11—C12	1.373 (4)
C2—H2B	0.9300	C11—H11A	0.9300
C3—C4	1.354 (5)	C12—H12A	0.9300
			
C14—S—C13	87.14 (13)	C12—C7—C8	120.7 (3)
C7—O—C4	119.3 (2)	C12—C7—O	116.0 (3)
C13—N1—N2	113.8 (2)	C8—C7—O	123.2 (3)
C14—N2—N1	112.0 (2)	C7—C8—C9	119.2 (3)
C14—N3—H3A	120.0	C7—C8—H8A	120.4
C14—N3—H3B	120.0	C9—C8—H8A	120.4
H3A—N3—H3B	120.0	C8—C9—C10	121.1 (3)
C6—C1—C2	119.2 (4)	C8—C9—H9A	119.4
C6—C1—H1B	120.4	C10—C9—H9A	119.4
C2—C1—H1B	120.4	C9—C10—C11	118.4 (3)
C1—C2—C3	121.2 (4)	C9—C10—C13	121.3 (3)
C1—C2—H2B	119.4	C11—C10—C13	120.3 (3)
C3—C2—H2B	119.4	C12—C11—C10	120.7 (3)
C4—C3—C2	119.0 (4)	C12—C11—H11A	119.6
C4—C3—H3C	120.5	C10—C11—H11A	119.6
C2—C3—H3C	120.5	C11—C12—C7	119.9 (3)
C3—C4—C5	120.6 (3)	C11—C12—H12A	120.1
C3—C4—O	119.5 (3)	C7—C12—H12A	120.1
C5—C4—O	119.9 (3)	N1—C13—C10	124.5 (3)
C4—C5—C6	119.2 (4)	N1—C13—S	113.1 (2)
C4—C5—H5A	120.4	C10—C13—S	122.4 (2)
C6—C5—H5A	120.4	N2—C14—N3	125.0 (3)
C1—C6—C5	120.7 (4)	N2—C14—S	113.9 (2)
C1—C6—H6A	119.6	N3—C14—S	121.1 (2)
C5—C6—H6A	119.6		
			
C13—N1—N2—C14	1.2 (4)	C9—C10—C11—C12	−0.7 (5)
C6—C1—C2—C3	−0.1 (7)	C13—C10—C11—C12	179.2 (3)
C1—C2—C3—C4	−0.2 (7)	C10—C11—C12—C7	0.4 (5)
C2—C3—C4—C5	−0.3 (6)	C8—C7—C12—C11	0.5 (5)
C2—C3—C4—O	−176.4 (3)	O—C7—C12—C11	−178.2 (3)
C7—O—C4—C3	−103.8 (4)	N2—N1—C13—C10	178.9 (3)
C7—O—C4—C5	80.0 (4)	N2—N1—C13—S	−0.7 (3)
C3—C4—C5—C6	1.0 (6)	C9—C10—C13—N1	178.9 (3)
O—C4—C5—C6	177.1 (4)	C11—C10—C13—N1	−1.0 (4)
C2—C1—C6—C5	0.8 (7)	C9—C10—C13—S	−1.6 (4)
C4—C5—C6—C1	−1.3 (7)	C11—C10—C13—S	178.6 (2)
C4—O—C7—C12	−166.5 (3)	C14—S—C13—N1	0.1 (2)
C4—O—C7—C8	14.8 (5)	C14—S—C13—C10	−179.6 (2)
C12—C7—C8—C9	−1.0 (6)	N1—N2—C14—N3	−179.2 (3)
O—C7—C8—C9	177.6 (3)	N1—N2—C14—S	−1.2 (3)
C7—C8—C9—C10	0.7 (5)	C13—S—C14—N2	0.6 (2)
C8—C9—C10—C11	0.2 (5)	C13—S—C14—N3	178.7 (3)
C8—C9—C10—C13	−179.7 (3)		

### Hydrogen-bond geometry (Å, °)

**Table:**

*D*—H···*A*	*D*—H	H···*A*	*D*···*A*	*D*—H···*A*
N3—H3A···N2^i^	0.86	2.21	3.042 (4)	162
N3—H3B···N1^ii^	0.86	2.26	3.094 (3)	163
C9—H9A···S	0.93	2.72	3.133 (4)	108

Symmetry codes: (i) −*x*, −*y*, −*z*+1; (ii) −*x*, *y*+1/2, −*z*+1/2.
